# Myopic Laser-Assisted Subepithelial Keratectomy (LASEK) outcomes using three different excimer laser platforms: a retrospective observational study

**DOI:** 10.1186/s12886-019-1214-y

**Published:** 2019-10-15

**Authors:** Isabel Rodríguez-Pérez, Juan Gros-Otero, Miguel A. Teus, Rafael Cañones, Montserrat García-González

**Affiliations:** 1 Clínica Novovisión Madrid, Paseo de la Castellana 54, 28046 Madrid, Spain; 2 European University of Madrid, Madrid, Spain; 3 Clínica Rementería, Madrid, Spain; 4 Hospital Universitario “Príncipe de Asturias”, University of Alcalá, Alcalá de Henares, Madrid, Spain

**Keywords:** Myopia, LASEK, Excimer laser, Mitomycin C

## Abstract

**Background:**

To compare the visual and refractive outcomes after myopic LASEK using three different excimer lasers and standardized surgical and mitomycin C (MMC) application protocols.

**Methods:**

In this retrospective, observational cohort study, we examined 122 eyes treated with Allegretto, 135 eyes treated with Esiris and 137 eyes treated with Technolas excimer lasers. All eyes were treated under the same surgical protocol, and a standardized MMC dosage was used. The three groups were refraction-matched, and both visual and refractive outcomes were evaluated at 1 and 7 days and 1 and 3 months after surgery.

**Results:**

At 3 months postsurgery, Allegretto provided significantly better outcomes than Esiris and Technolas in terms of postoperative uncorrected distance visual acuity (UDVA) (1.11 ± 0.2 vs 1.01 ± 0.2 vs 0.98 ± 0.2) (*P* = 0.0001), corrected distance visual acuity (CDVA) (1.13 ± 0.2 vs 1.10 ± 0.1 vs 1.04 ± 0.2) (*P* = 0.0001), residual sphere (− 0.01 ± 0.2 vs + 0.29 ± 0.7 vs + 0.27 ± 0.6) (*P* = 0.0001), and efficacy index (0.99 ± 0.2 vs 0.90 ± 0.2 vs 0.91 ± 0.2) (*P* = 0.0004).

**Conclusions:**

We found slightly better visual and refractive outcomes in the Allegretto group at 3 months post-op after LASEK with MMC to correct myopia.

## Background

Both laser in situ keratomileusis (LASIK) and surface ablation (SA) have been proven to be safe, effective and predictable procedures, becoming the gold standard for myopia correction [1–3]. LASIK flap creation has been suggested to induce higher corneal biomechanical changes [4, 5] and higher order aberrations (HOAs) than SA [6, 7]. In addition, the use of corneal wound healing modulators and the technological development of last excimer laser platforms have peaked interest in SA techniques in recent years.

The introduction of mitomycin C (MMC) for corneal wound healing modulation after SA procedures has led to an increase in the treatment range, thus being comparable to LASIK indications. The use of MMC in SA results in a lower incidence of haze [8, 9], higher predictability [10, 11] for low and moderate myopia and an improvement of long-term refractive stability [12].

The development of new excimer laser devices with advanced ablation profiles, faster ablation rates, more accurate eye trackers, and lower stromal ablations for equal refractions in older excimer lasers has led to better refractive outcomes [13], providing similar results for myopia correction in SA and LASIK [1, 2]. Recently, significant interest has been shown in the outcome variability among different laser platforms. Refractive outcomes and/or biological corneal responses might be different between excimer laser platforms due to differences in ablation profiles, energy levels, laser energy stability, etc. The available excimer laser devices include the WaveLight Allegretto® (WaveLight Laser Technologie AG, Erlangen, Germany), the Esiris® (Schwind eye-tech-solutions Gmbh & Co, Kleinostheim, Germany) and the Technolas® (Bausch & Lomb Surgical, Claremont, CA) excimer lasers. Several comparative studies have been published describing excellent results when myopic LASIK is performed with different excimer laser platforms [14–18], including these three devices [19–23].

Although the Allegretto®, Esiris® and Technolas® platforms have been widely studied individually in several publications [24–30], few comparative studies among them have been published [31–35]. Furthermore, only four of the previous studies were designed to compare visual and refractive results [31–34]. In those comparisons, the heterogeneous age ranges [31], the different ablation profiles for each laser studied [32–34], the lack of MMC protocol in some cases [35] or the restriction of MMC protocols for the same degree of depth ablation [34] should be considered as certain biases that could have influenced the final results.

Thus, we compared the visual and refractive outcomes obtained with three different excimer laser platforms used for myopic laser-assisted subepithelial keratectomy (LASEK) correction in a young adult population (≤ 40 years old), performed by the same surgeon, using conventional ablation profiles in all devices and following the same surgical protocol in all treated eyes.

## Material and methods

This study is a retrospective cohort study of 394 eyes from consecutive patients younger than 40 years who underwent LASEK for the correction of myopia with or without astigmatism between 2005 and 2014.

Patients with unstable refraction, keratoconus suspects (defined as any even mild localized steepening observed with Placido corneal topography or slight bowing of the posterior corneal surface detected by corneal tomography), prior ocular surgeries, or systemic diseases that could alter refractive or visual outcomes were excluded.

The preoperative examination, which included corrected distance visual acuity (CDVA) (Nidek autochart projector CP 670, Nidek, Gamagori, Japan), manifest and cycloplegic refraction, ultrasound corneal pachimetry (DGH 5100 contact pachymeter, DHG Technology Inc., Exton, PA; OcuScan RXP, Alcon Laboratories, Inc., Fort Worth, TX), topography/tomography and keratometry (Dicon CT200, Vismed Inc., San Diego, CA; CSO Construzione Strumenti Oftalmici, Italy), mesopic infrared pupillometry (Colvard Pupillometer, Oasis 78 Medical Inc., Glendora, CA), slit-lamp biomicroscopy, Goldmann tonometry and funduscopy, was performed by a masked observer.

All patients provided written informed consent, and institutional review board approval was obtained (regional committee of clinical research of the Community of Madrid. REF 216/3). The study was performed in accordance with the tenets of the Declaration of Helsinki.

### Surgical technique

An experienced surgeon (M.A. T) performed all procedures in private practice ophthalmic clinics.

A povidone-iodine solution 5% was applied on the eyelid and conjunctiva before the sterile surgical drape and an eyelid rigid speculum was positioned. All surgeries were performed under topical anaesthesia (lidocaine 2%). A 20% ethanol solution diluted in balanced salt solution (BSS) was instilled for 40 s inside an 8.5-mm corneal trephine (ASICO, Westmont, IL) centred on the pupil. The ethanol solution was eliminated with a cellulose sponge (Merocel®, Medtronic Ophthalmics) and gently rinsed with a cannula connected to a BSS syringe. Once the edges of the epithelial flap were dried with a cellulose sponge, the flap was peeled back with a crescent blade (Alcon Surgical, Orlando, FL), leaving a superior hinge (12-o’clock position). The stromal ablations were performed with the following excimer lasers depending on the date each device remained at the facilities: Wavelight Allegretto 400 Hz®, WaveLight Laser Technologies AG, hereafter “A device”, Esiris®, Schwind Eye Tech Solutions, hereafter “E device” or Technolas 517c®, Bausch & Lomb Surgical, excimer lasers, hereafter “T device”. The A device used 0.95 mm spot separation; fluence: 200 mJ/cm^2^; and repetition rate: 400 Hz. The E device used 0.95 mm spot separation; fluence: 650 mJ/cm^2^; and repetition rate: 200 Hz. The T device used 0.95 mm spot separation; fluence: 600 mJ/cm^2^; and repetition rate: 50 Hz. A 6–7.5 mm optical zone (larger than or equal to the mesopic pupillary size) was ablated using a conventional ablation algorithm (non-customized) in all treated eyes, according the manufacturer’s recommendations.

After stromal ablation, a sponge soaked in MMC 0.02% was applied over the stromal bed for 15 s for every 50-μm ablation depth. For ≤50-μm ablation depths, 15 s of MMC were applied, avoiding leakage to the epithelial flap and the limbus. The programmed spherical ablation was 10% less than the intended correction to avoid overcorrection caused by MMC. The residual stromal bed was gently rinsed with balanced salt solution, and the epithelial flap was repositioned over the stromal bed. A therapeutic soft contact lens (AcuVue; Johnson & Johnson Vision Care, Inc., Jacksonville, FL) was carefully placed on the eye, and antibiotic drops (ciprofloxacin 3 mg/mL, Oftacilox®, Alcon Cusí, Barcelona, Spain) and nonsteroidal anti-inflammatory drops (ketorolac trometamol 5 mg/mL, Acular®, Allergan, Madrid, Spain) were instilled before the speculum was removed.

### Postoperative follow-up

Ciprofloxacin 3 mg/mL and steroid drops (dexamethasone alcohol 1 mg/mL, Maxidex®, Alcon Cusí) were applied four times daily during the first postoperative week. The therapeutic contact lens was removed at 1 week after surgery. The steroid drop dosage was tapered over the next 2 months as follows: three times daily for the first month, twice daily for the following 15 days and once daily for the last 15 days. Preservative-free artificial tears were applied as needed.

All patients were examined at 1-day, 1-week, and 1- and 3-month postoperative visits by two experienced optometrists, who recorded, under standardized registration, the uncorrected distance visual acuity (UDVA) and corrected visual acuity (CDVA) in the same room under the same illumination adjusted to mesopic conditions. At the three-month visit, a complete ocular examination was performed, including manifest residual refraction, CDVA and topography.

### Statistical analysis

Statistical analysis was performed with the “Statview SE + Graphics”™ (Abacus Concepts Inc., Berkeley, CA) program and a Macintosh PowerBook 1400cs/117 personal computer (Apple Computer Inc., Cupertino, CA, USA). A decimal scale was used for visual acuity measurements and converted to LogMAR quotation using a conversion chart for statistical analysis. The data included the mean, standard deviation, standard error and range. The Kolmogorov-Smirnov test was used to check normality of the distribution, and a factorial ANOVA test was used for analysis. In addition, an intra-group linear regression analysis was performed. A 95% confidence interval was set up, and a *P* value < 0.05 was considered statistically significant.

## Results

This study included 394 consecutive myopic eyes that were divided into three refraction-matched groups: 122 eyes treated with device A, 135 eyes treated with device E, and 137 eyes treated with device T. The preoperative sphere and cylinder were matched in ±0.50 D between groups.

Preoperative sphere in all groups was ≤ − 11.00 D and cylinder was ≤ − 4.50 D. Some statistically significant differences were found in terms of keratometry, pachymetry and age due to the large sample size of this study. Nevertheless, these differences were small and not clinically relevant. Preoperative data are shown in Table 1.

**Table 1:** Preoperative data for the 394 eyes treated with Allegretto, Esiris and Technolas excimer lasers in a follow-up period of 3 months after myopic LASEK with the adjuvant use of mitomycin C

Parameter	ALLEGRETTO	ESIRIS	TECHNOLAS	*P*-value^a^
Eyes	122	135	137	
Sphere (D) (≤ − 11 D)	−4.10 ± 2.16	− 4.16 ± 2.20	−4.23 ± 2.18	0.9
Cylinder (D) (≤ −4.5 D)	−0.90 ± 0.96	− 0.90 ± 0.86	− 0.89 ± 0.81	0.9
CDVA (LogMAR)	−0.04 ± 0.06	− 0.04 ± 0.05	− 0.03 ± 0.07	0.06
CDVA (Decimal)	1.12 ± 0.13	1.11 ± 0.12	1.08 ± 0.10	0.05
CCT (μm)	524 ± 26.00	514 ± 31.20	520 ± 28.00	0.03
Keratometry K1 (D)	43.12 ± 1.50	43.77 ± 1.50	43.65 ± 1.50	0.0005
Keratometry K2 (D)	44.10 ± 1.60	44.90 ± 1.60	44.60 ± 1.50	0.0001
Age (years)	28.60 ± 4.50	30.40 ± 5.50	31.80 ± 7.40	0.0002

*CDVA* corrected distance visual acuity, *CCT* central corneal thickness, *D* dioptres

^a^Factorial ANOVA test

**Fig. 1:**
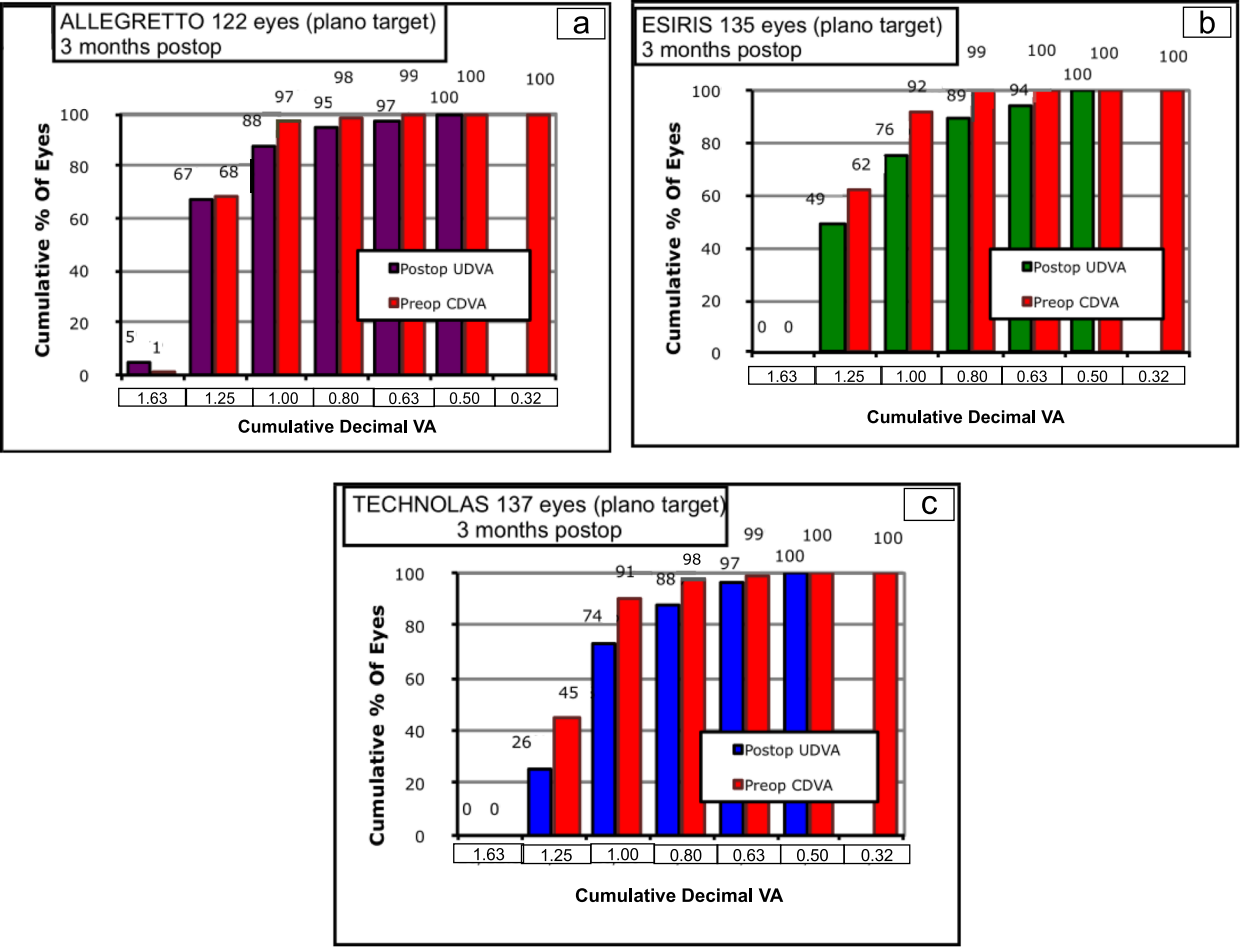
Cumulative histogram of three-month uncorrected distance visual acuity after myopic LASEK with MMC of 394 eyes treated with the Allegretto, Esiris and Technolas excimer lasers. LASEK = Laser-assisted subepithelial keratomileusis; MMC = Mitomycin C; VA = Visual acuity. **a**- Allegretto, **b**- Esiris, **c**-Technolas

At 1 day, 1 week and 1 month postoperatively, UDVA showed no statistically significant differences among groups, in both decimal and LogMAR notations. However, at the three-month visit, UDVA showed statistically significant better results in the A device group compared to both the E and T device groups (*P* = 0.0001), in both decimal and LogMAR notations (Table 2). Cumulative UDVA is shown in Fig. 1.

**Table 2:** UDVA evolution up to three months after LASEK with MMC of 394 myopic eyes treated with Allegretto, Esiris and Technolas excimer lasers

Parameter Mean ± SD	Follow-up visit	ALLEGRETTO	ESIRIS	TECHNOLAS	*P*-value^a^
Eyes		(*n* = 122)	(*n* = 135)	(*n* = 137)	
UDVA (Decimal)	1 day	0.64 ± 0.33	0.63 ± 0.24	0.57 ± 0.21	0.2
	1 week	0.72 ± 0.21	0.80 ± 0.24	0.81 ± 0.61	0.4
	1 month	0.94 ± 0.23	0.92 ± 0.17	0.88 ± 0.19	0.07
	3 months	1.11 ± 0.19	1.01 ± 0.21	0.98 ± 0.20	0.0001
UDVA (LogMAR)	1 day	0.29 ± 0.38	0.23 ± 0.20	0.27 ± 0.17	0.5
	1 week	0.16 ± 0.15	0.12 ± 0.17	0.13 ± 0.17	0.4
	1 month	0.04 ± 0.13	0.04 ± 0.10	0.06 ± 0.11	0.2
	3 months	−0.03 ± 0.10	0.09 ± 0.11	0.01 ± 0.10	0.0001

*UDVA* uncorrected distance visual acuity, *SD* standard deviation

^a^Factorial ANOVA test

Three months postoperatively, statistically significant better results in CDVA were obtained, in decimal notation, with the A device and E device compared to the T device (*P* = 0.0001), whereas in LogMAR notation, these differences were not detected among the groups, although a slight tendency to significance was noted between the E device and the T device (*P* = 0.05) (Table 3).

**Table 3:** Three-months postop outcomes after LASEK with MMC of 394 myopic eyes treated with Allegretto, Esiris and Technolas excimer lasers

Parameter Mean ± SD	ALLEGRETTO	ESIRIS	TECHNOLAS	*P*-value^a^
Eyes	122	135	137	
Residual Sphere (D)	−0.01 ± 0.24	0.29 ± 0.65	0.27 ± 0.58	0.0001
Residual Cylinder (D)	−0.06 ± 0.32	−0.37 ± 0.53	− 0.26 ± 0.53	0.0001
CDVA (LogMAR)	−0.02 ± 0.17	−0.04 ± 0.10	− 0.01 ± 0.08	0.05
CDVA (Decimal)	1.13 ± 0.15	1.10 ± 0.13	1.04 ± 0.17	0.0001
Efficacy index	0.99 ± 0.16	0.90 ± 0.18	0.91 ± 0.18	0.0004
Safety index	1.00 ± 0.12	1.07 ± 0.94	0.90 ± 0.14	0.03
Change in lines of CDVA	−0.001 ± 0.11	−0.007 ± 0.10	− 0.025 ± 0.11	0.06

*CDVA* corrected distance visual acuity, *D* dioptres, *SD* Standard deviation

^a^Factorial ANOVA test

At the last follow-up visit, residual sphere and cylinder showed statistically significant differences among groups. Thus, the A device provided a lower and statistically significant residual sphere (*P* = 0.0001) and a lower and statistically significant residual cylinder (*P* = 0.0001) than those of the E and T device groups (Table 3).

**Fig. 2:**
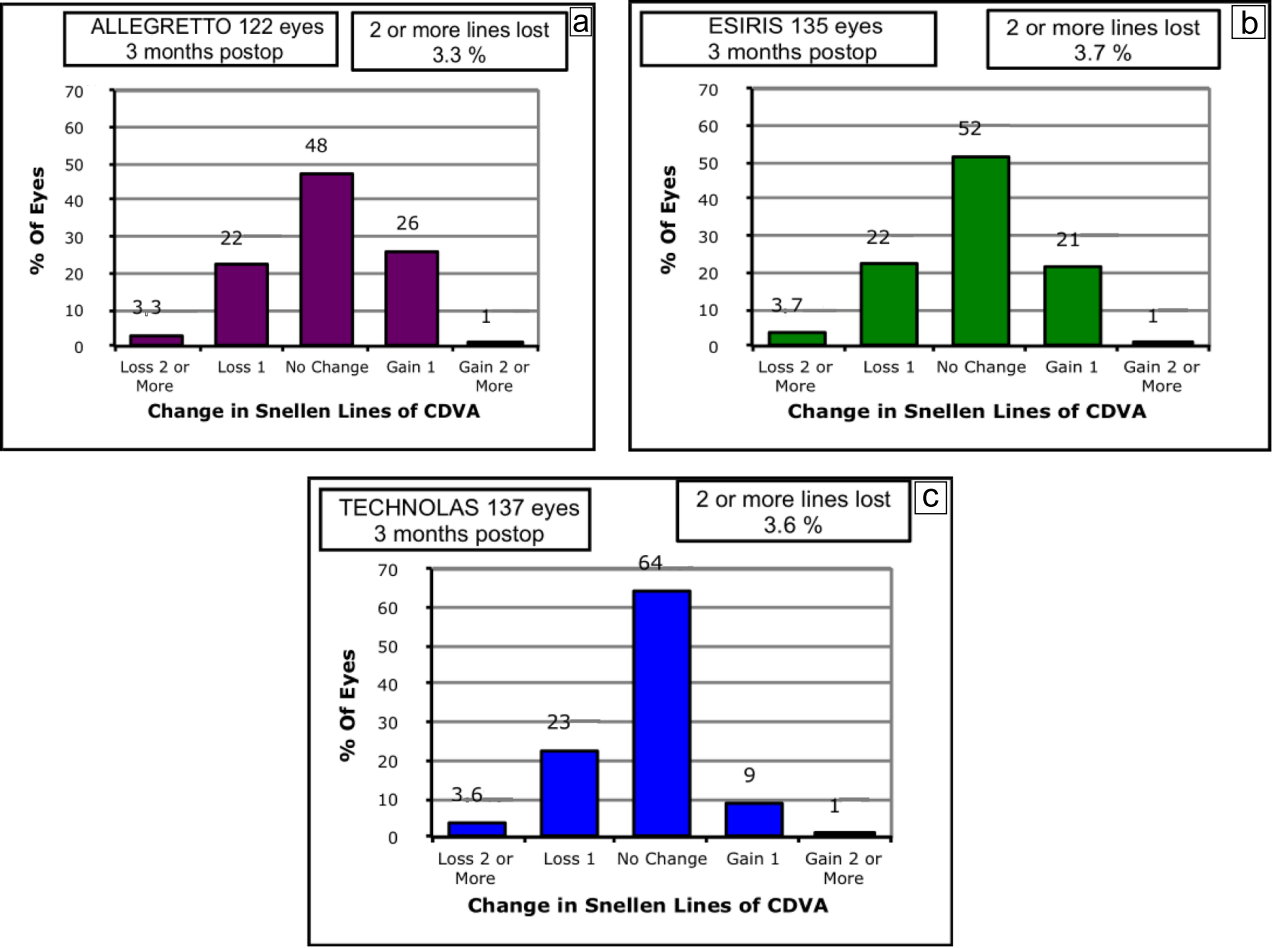
Changes in lines of corrected distance visual acuity at 3 months after myopic LASEK with MMC of 394 eyes treated with Allegretto, Esiris and Technolas excimer lasers. LASEK = Laser-assisted subepithelial keratomileusis; MMC = Mitomycin C; CDVA = Corrected distance visual acuity. **a**- Allegretto, **b**- Esiris, **c**-Technolas

The efficacy index (defined as the postoperative UDVA/preoperative CDVA) showed better outcomes in the A device group than in the E and T device groups. These differences were statistically significant (*P* = 0.0004) (Table 3). The percentage of cumulative UDVA at the three-month visit is shown in Fig. 1. The safety index (defined postoperative CDVA/preoperative CDVA) showed statistically better results between the A device or E device group and the T device group (*P* = 0.03), whereas significant differences between the A device and E device groups were not detected (Table 3). Changes in lines of CDVA are shown in Fig. 2. The mean changes in lines of preoperative and postoperative CDVA are shown in Table 3.

**Fig. 3:**
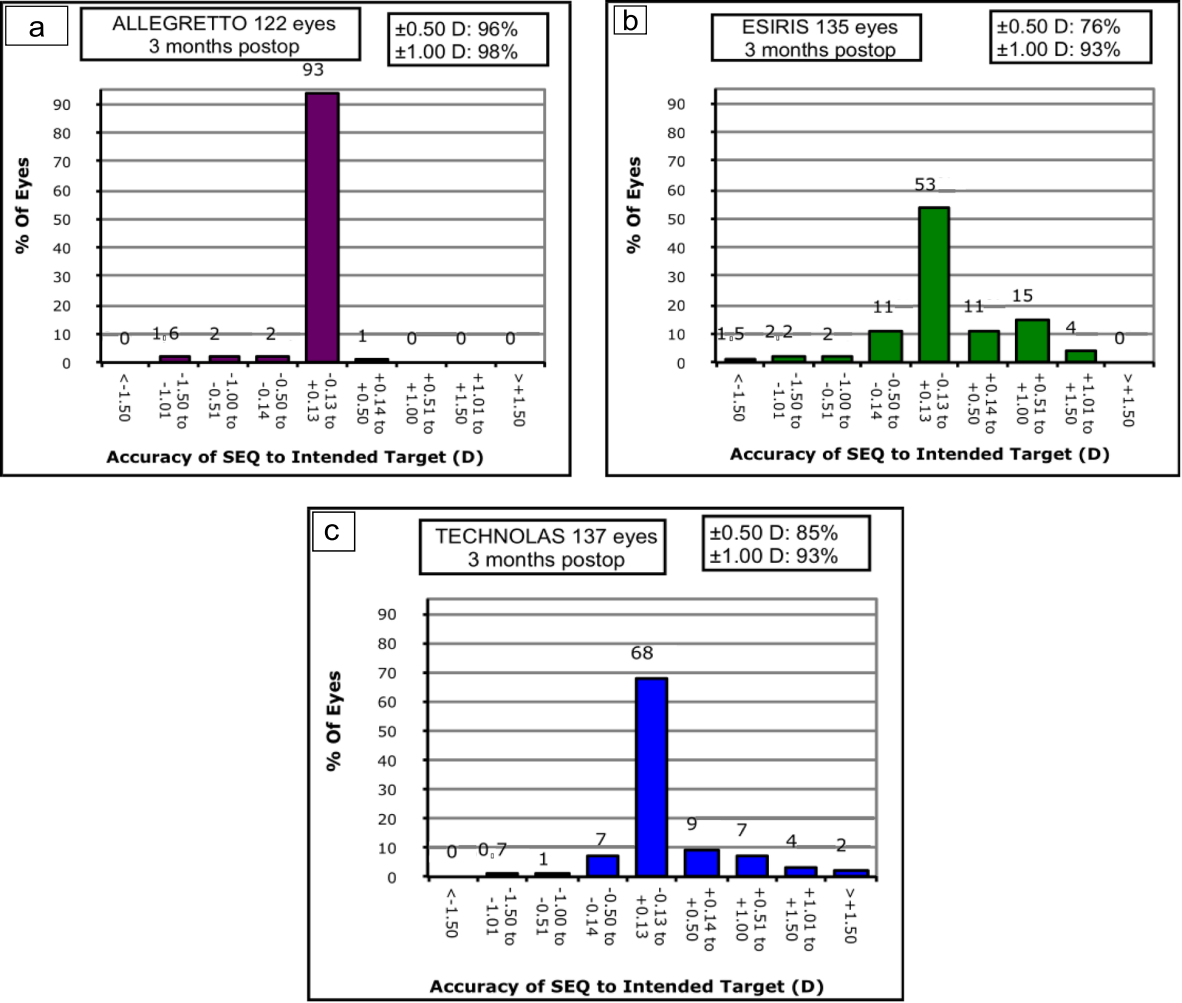
Three-month predictability (SE ± 0.50 D and ± 1.00 D) after LASEK with MMC in the Allegretto, Esiris and Technolas groups for myopia correction. LASEK = Laser-assisted subepithelial keratomileusis; MMC = Mitomycin C; SE = Spherical Equivalent. **a**- Allegretto, **b**- Esiris, **c**-Technolas

**Fig. 4:**
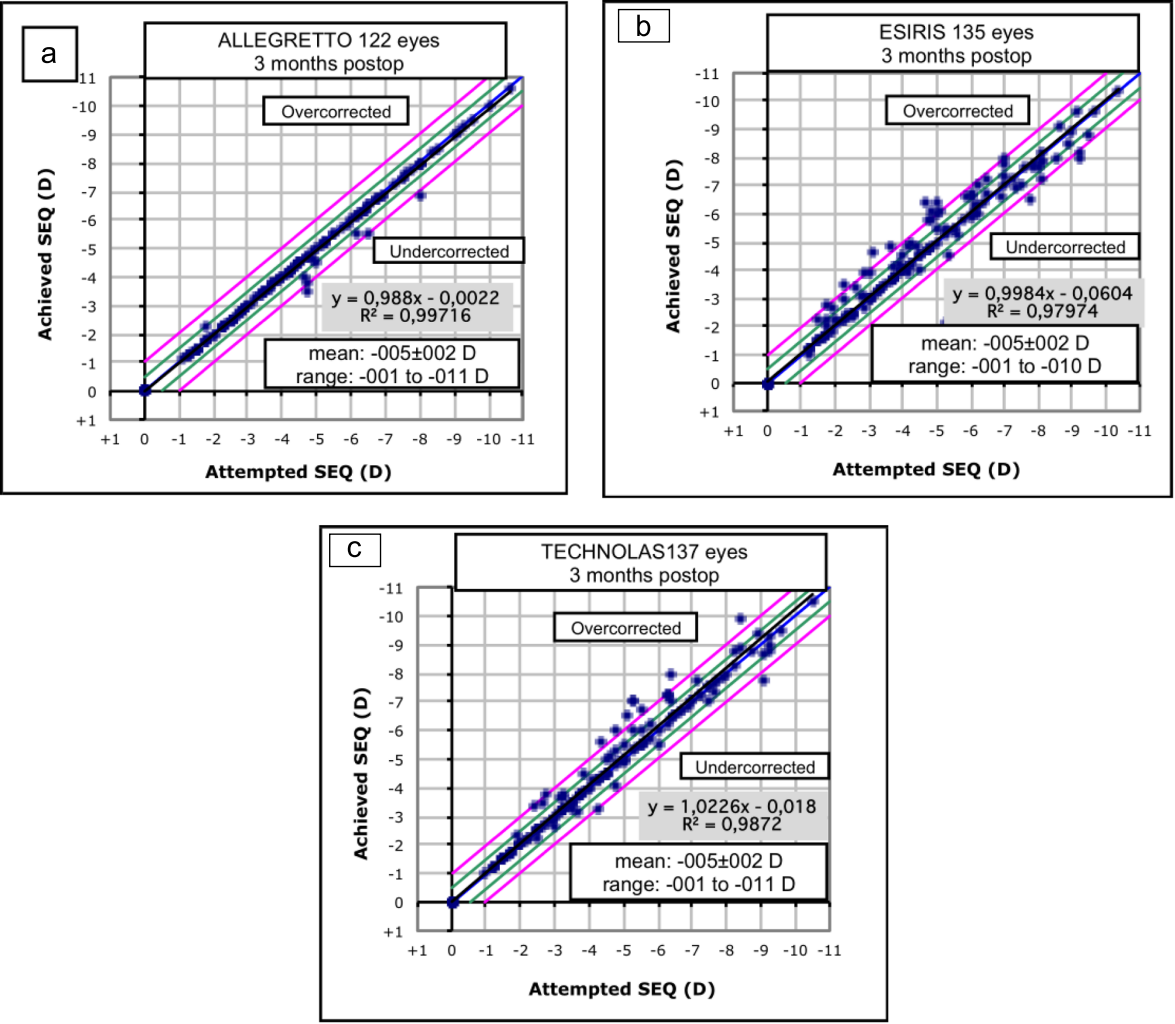
Attempted versus achieved spherical equivalent refraction scatterplots at 3 months after LASEK with MMC for myopia correction in the Allegretto, Esiris and Technolas groups. The linear regression equation and coefficient of determination (r^2^) are displayed. LASEK = Laser-assisted subepithelial keratomileusis; MMC = Mitomycin C. **a**- Allegretto, **b**- Esiris, **c**-Technolas

Predictability for residual spherical equivalent (SE) ± 0.50 D showed statistically significant differences among groups (*p* = 0.0001) (Fig. 3). Predictability for residual SE ± 1.00 D is shown in Fig. 3. No differences were detected among groups (*p* = 0.08). Linear regression analysis showed a high, positive and statistically significant correlation between preoperative SE and the effectively corrected refraction in all evaluated groups (Fig. 4).

No intraoperative or postoperative complications were found in any group.

## Discussion

Marginally better predictability and efficacy results were noted with device A at the three-month follow-up visit. However, we found that LASEK surgery is safe, predictable and effective using any of the three excimer laser platforms studied. Although significant differences were detected among the three devices, it should be noted that this difference is so small that it has no clinical impact.

All groups showed initial slow UDVA recovery, as previously described [36]. No differences were found until the first month follow-up visit, but at the 3 month follow-up visit, the A device patients showed significantly better UDVA and efficacy index results. Although variations in the energy level of the laser beam, which are common when using the first excimer devices during the treatments, could affect the predictability, these deficiencies have been improved considerably by manufacturers in the modern laser platforms, achieving optimal energy stability and very precise nomograms. In addition, a higher time of exposure of the stroma alters the corneal hydration, and thus, a greater number of treatment interruptions because of eye movements and primitive eye-tracking systems could also affect the predictability of the ablation. These improvements, together with the greater speed of the laser, could also justify the differences we observed in our study.

Published series for SA using the A device showed comparable results to ours, even with older excimer laser versions (A device 200 Hz) [24, 29, 30]. However, slightly worse results in UDVA were found using the A device 400 Hz in other published series [26]. Isolated E device series showed results comparable to ours, with subtle differences among groups [25, 27]. Prior isolated T device series also obtained similar results [28]. The slight differences observed might be related to higher age range recruitment requirements or the different refraction ranges studied, in addition to the lack of certain data (i.e., excimer laser version or age range), which may hinder the analysis.

Five studies were found that compared SA refractive results with different excimer lasers [31–35], but none of these studies included the three excimer laser platforms examined in our study. Nassiri et al. published two different PRK series [32, 33] in which slightly better UDVA was obtained with the A device compared to the T device, as found in the present study. Compared with VISX Star S4 [34, 35] the A device obtained better UDVA. In these studies, the authors described MMC protocols, refraction ranges and ablation profiles that were different from ours and among these studies, which is problematic as these difference interfere with the proper comparison of these studies. As previously described, using the same surgical protocol, standard ablation procedures for each platform and the same MMC protocol, those biases were diminished.

No statistically significant differences were found among groups in preoperative corrected distance visual acuity (CDVA) in decimal and LogMAR charts. The three-month postoperative CDVA was statistically better for the A device and E device groups than for the T device group, whereas in LogMAR notation, these differences were not detected among the groups. Similar results were found in the safety index analysis, and the number of eyes with vision lines lost was worse with T device data than with data from the other devices. The A device and E device results were similar to previous publications [25–27], but our T device results were worse than other results [32, 28]. It Some degree of epithelial alteration, subclinical haze, etc., improves the visual results of surface ablation, even at 3 months after surgery. Nevertheless, since the purpose of the current paper was not to establish the efficacy and safety of surface ablation but to compare the results obtained with different laser platforms, evaluating all results at the same point in the postoperative examination is mandatory, even if the visual results are still not definitive.

In the last follow-up visit of our study, the spherical and cylindrical residual refraction with the A device was different than those with the T and E devices, in both paired comparison and regression analyses. Regarding spherical residual refraction, the A device obtained near to plano refraction with myopic tendency, whereas the E and T devices overcorrected. With cylindrical residual refraction, the A device was also near the plano, with E and T devices undercorrecting. Regression analysis showed low residual refraction dispersion, despite planned treated refraction in eyes treated with the A device, whereas higher dispersion was found both in the E and T devices.

Predictability (SE ±0.50 D) was also better for the A device than for the other two devices. Knowing the optimal predictability results of every excimer laser platform available and its improvement compared to initial models [37, 38], we suggest that the predictability analysis might circumscribe to the ±0.50 D analysis, thus overcoming the prior SE measurement of ±1.00 D.

When we designed this study, our goal was to unmask the subtle differences between excimer laser platforms by creating an ideal surgical workflow where the possible biases were diminished as much as possible, although some of these platforms might not be commercially available at the present time. We used a standardized surgical procedure by a single surgeon to avoid surgical biases. Refraction-matched patients were recruited to avoid postoperative refractive results biases among groups. Finally, a standard MMC protocol [3, 8, 11, 39, 40] was used for biological response homogenization.

Different biological responses that exceed the MMC effect might be expected for each excimer laser studied due to their technical differences. While we did not find haze in any studied group, we propose that the biological homogenization induced by MMC affects all groups in the same way. For these reasons, we can hypothesize that the differences found are real and related to the specific technical features of each excimer laser platform studied.

The studied excimer laser platforms have also been studied in LASIK surgeries [19–23]. Given the known differences between LASIK and SA, the direct comparison of our results with previous publications exceeds the aim of our work. Table 4 shows the results of previous studies on similar laser platforms for SA and LASIK procedures.

**Table 4:** Comparative publications among different excimer platforms for Anterior Surface Ablation/LASIK performance

	Study design	Treatment	MMC 0.02%	UDVA decimal	UDVA LogMAR	Efficacy Index	Residual Sphere	Residual Cylinder	CDVA decimal	CDVA LogMAR	Safety Index	Change in CDVA lines preop/postop	SE ±0.5 D	SE ±1 D	Correlation preopSE/ Corrected refraction
ANTERIOR SURFACE ABLATION															
Our Study	Retrospective study. 394 eyes. ≤ 40 y.o. Full range myopia 3-months follow-upU Allegretto 400 Hz vs Esiris vs Technolas 217c.	Standard (no customized)	MMC: 15 s each 50 μm	*Allegretto better than others	*Allegretto better than others	*Allegretto better than others	*Allegretto better than others	*Allegretto better than others	*Allegretto better than others	No differences were found	*Technolas worse than others	No differences were found	*Allegretto better than others	No differences were found	Strong correlation in the 3 groups
[32] Nassiri et al. JCRS. 2011;37:1858–64.	Prospective study 131 eyes. ≤ 40 y.o. Low to moderate myopia. 3-month follow-up Allegretto 400 Hz vs Technolas 217z.	WFG vs Planoscan	MMC: 10–25 s > 70 μm	Allegretto better than Technolas			No differences were found	No differences were found	Allegretto better than Technolas						
[33] Nassiri et al. JRS. 2015;31:683–90.	Prospective study. 151 eyes. ≤ 45 y.o. Moderate myopia. 3-month follow-upU Allegretto 400 Hz vs Technolas 217z	WFO vs Tissue-saving	MMC: 10–25 s > 70 μm	Allegretto better than Technolas			No differences were found	No differences were found	20/20 (Snellen) in both groups						
LASIK															
[20] Han et al. Clin Ophthalmol. 2012; 6:1159–68.	Retrospective study. 442 eyes. ≤ 52 y.o. Full range myopia 3-month follow-upU Allegretto 400 Hz vs Technolas 217z	WFO vs Planoscan Flap creation: Visumax 500 kHz			Allegretto better than Technolas	No differences were found	Allegretto better than Technolas	*Allegretto better than Technolas		No differences were found	No differences were found		No differences were found	No differences were found	
[19] Mearza et al. J Refract Surg. 2008;24:885–90.	Prospective study 44 eyes. **≤** 61 y.o. Full range myopia and hyperopia Allegretto eye-Q vs Esiris	Q-value vs aspheric standard Flap creation: Moria M2		No differences in myopia subgroup were found		No differences in total group were found	No differences in myopia subgroup were found	No differences in myopia subgroup were found	No differences in total group were found		No differences in total group were found		No differences in total group were found	No differences in total group were found	

*UDVA* uncorrected distance visual acuity; *CDVA* corrected distance visual acuity, *D* dioptres, *MMC* mitomycin C, *SE* spherical equivalent, *y.o.*: years old; *statistically significant differences (*P* < 0.05)

Certain possible biases in our results analysis are avoided by delimiting the recruitment maximum age (under 40) and by matching the spherical and cylindrical refraction; the preoperative significant differences obtained in the keratometry and pachymetry values were small and not clinically relevant.

## Conclusions

In conclusion, we found subtle postoperative differences favouring the Allegretto excimer laser after myopic LASEK surgery with a standardized surgical and MMC protocol when studying matched populations. However, it should be noted that these differences are so small that they have no clinical impact.

## Data Availability

The datasets used and/or analysed during the current study are available from the corresponding author on reasonable request.

## Abbreviations

A: Allegretto excimer laser
BSS: Balanced salt solution
CDVA: Corrected distance visual acuity
D: Dioptre
E: Esiris excimer laser
LASEK: Myopic laser-assisted subepithelial keratectomy
LASIK: Laser in situ keratomileusis
MMC: Mitomycin C
SA: Surface ablation
SE: Spherical equivalent
T: Technolas excimer laser
UDVA: U
